# Therapeutic Effects of Quercetin on Inflammation, Obesity, and Type 2 Diabetes

**DOI:** 10.1155/2016/9340637

**Published:** 2016-11-28

**Authors:** Shuang Chen, Hongmei Jiang, Xiaosong Wu, Jun Fang

**Affiliations:** ^1^College of Bioscience and Biotechnology, College of Animal Science and Technology, Hunan Agricultural University, Changsha 410128, China; ^2^Hunan Province University Key Laboratory for Agricultural Biochemistry and Biotransformation, Hunan Agricultural University, Changsha 410128, China; ^3^Hunan CoInnovation Center for Ultilization of Botanical Functional Ingredients, Changsha 410128, China

## Abstract

In previous studies, abdominal obesity has been related to total low-grade inflammation and in some cases has resulted in insulin resistance and other metabolism related disorders such as diabetes. Quercetin is a polyphenol, which is a derivative of plants, and has been shown* in vitro *as well as in a few animal models to have several potential anti-inflammatory as well as anticarcinogenic applications. The substance has also been shown to aid in the attenuation of lipid peroxidation, platelet aggregation, and capillary permeability. However, further research is called for to gain a better understanding of how quercetin is able to provide these beneficial effects. This manuscript reviewed quercetin's anti-inflammatory properties in relation to obesity and type 2 diabetes.

## 1. Introduction

The world has seen a rapid increase in the incidence of diabetes generally, and type 2 diabetes has become the most common metabolic disease globally. This has been in part due to a growth in obesity, especially excess visceral adiposity, in addition to the associated “metabolic syndrome” which encompasses insulin resistance, hyperglycaemia, dyslipidaemia, and hypertension [1].

Inflammation occurs in response to various pathological stimuli and tissue injury, and chronic inflammation and the activation of the immune system may be mainly responsible for the process of obesity-related metabolic diseases such as type 2 diabetes [2, 3]. Key characteristics of type 2 diabetes include insulin secretion defects and insulin resistance in liver, adipose tissue, and skeletal muscles. Diabetes and associated complications are a result of the inflammatory processes [4]. Interventional studies have confirmed the aspects of inflammation occurring within type 2 diabetes pathogenesis. Metformin and peroxisome proliferator-activated receptor- (PPAR-) *γ* agonist has been shown to use anti-inflammatory mechanisms to effectively lower the occurrence of type 2 diabetes and its related complications [5]. These studies further indicate that drugs aimed at hypoglycaemia may also have anti-inflammatory and therefore antidiabetic characteristics.

Unique biological elements of the flavonoid quercetin (found in fruits and vegetables) contain potential mental and physical health benefits. Among these are disease resistance; enhanced mental and physical performance; the ability to inhibit lipid peroxidation; stimulation of mitochondrial biogenesis; and other anti-inflammatory, antiviral, and antioxidant properties [6, 7].

This study will review the effects of quercetin as a dietary supplement. Quercetin's impact on inflammation, disease resistance, and overall health will be reviewed with the goal of summarising its main potential therapeutic applications. The data within the peer-reviewed literature are being considered within the study, which will be based on the cellular, molecular, and fundamental functions of possible therapy through the use of Quercetin.

## 2. Inflammatory Markers of Obesity

The development of insulin resistance, diabetes, and a higher possibility of contracting cardiovascular disease has been linked to obesity [8]. Examinations of overweight and obese adults have revealed altered circulating levels of inflammatory cytokines such as interleukin-1*β* (IL-1*β*), interleukin-6 (IL-6), tumour necrosis factor-alpha (TNF-*α*), or C-reactive protein (CRP) [9]. Although body fat generally has been correlated with serum levels of inflammatory proteins, abdominal obesity has shown a stronger correlation than either Body Mass Index (BMI) or total body fat [9], for all individuals, including those in good health.

Hermsdorff et al. associated abdominal fat accumulation with concentrations of IL-6, CRP, and complement factor C3 [10]. Some researches have demonstrated a correlation between the fats in abdomens and CRP concentration within individuals that are not obese. The evidence points to an increased risk associated with visceral adipose tissue vis-a-vis subcutaneous adipose tissue [11].

## 3. Inflammatory Markers of Type 2 Diabetes

Type 2 diabetes patients undertaking a more active lifestyle to reduce weight have been shown to exhibit improvement in a variety of factors. These factors include the count of white blood cell, CRP, serum amyloid A (SAA), and proinflammatory cytokines such as TNF-*α*, IL-6, and IL-1*β* [10, 12]. Additionally, irrespective of the level of insulin resistance and obesity at the outset, an independent risk factor for the development of type 2 diabetes seems to be subclinical chronic inflammation.

Among type 2 diabetes indicators, CRP measurement is a relatively inexpensive, standardised, and readily available measure. Other indicators described in prospective studies include the level of white blood cells, proinflammatory cytokines, chemokines, and numerous inflammatory biomarkers including fibrinogen and sialic acid [13–15]. Several prospective studies show high sensitivity-CRP (hs-CRP) levels are a good predictor of the future advancement of type 2 diabetes in nondiabetic individuals regardless of fat distribution and insulin resistance. A measurement of CRP that is incredibly sensitive has been created to accurately detect CRP at lower levels. There has been current meta-analysis and appraisal of CRP-related studies further demonstrated that higher levels of CRP are correlated with the increased risk for type 2 diabetes [16].

## 4. Dietary Sources of Quercetin

Flavonols, primarily in the form of glycosides, exist in part that is edible within numerous plants including leafy vegetables, many fruits, bulbs and tubers, herbs, spices, tea, and also wine [17]. Among these, quercetin has the highest amount of flavonol molecules. Furthermore, the majority of quercetin-type flavonols consumed consist of quercetin glycoside conjugates in which quercetin is related to 1-2 glucose residues. As a result, within the average diet there are relatively fewer quantities of quercetin aglycones [18].

Quercetin levels in food have been found to be impacted by growing conditions. For example, in the case of tomatoes, a higher quercetin aglycone level has been found in those organically grown as compared to those grown using traditional growing techniques.

Dietary consumption of quercetin differs across countries. Flavonoid daily intake (in which about 75% is quercetin) ranges from a low of 5 milligrams per day to a high of 80 milligrams. Key among the variables influencing the level is the amount of fruits and vegetables and tea consumed. On average, men as well as older individuals consume relatively lower levels of quercetin; and seasonal consumption (i.e., summer versus winter levels) showed no significant difference. Flavonol intake levels in the United States are about 13 milligrams per day for adults, with quercetin accounting for about 75% [19]. In northern China, quercetin intake is found to be only about 4.37 milligrams per day, with the chief flavonol crop being apples with 7.4%, followed by potatoes at 3.9%, and lettuce and oranges at 3.8% each [20]. Averages quercetin consumption in the Chinese city of Harbin followed a similar but more diverse blueprint: 4.43 milligrams per day, made up of apples (3.7%), potatoes (2.5%), celery (2.2%), eggplant (2.2%), and* Actinidia* (1.6%) [21]. Japanese average and median intake are much higher at 16.2 and 15.5 milligrams per day, respectively.

## 5. Anti-Inflammatory Effect of Quercetin

Extracts from* Mexican oregano* have demonstrated anti-inflammatory properties through decreasing the production of reactive oxygen species (ROS) and nitric oxide (NO) [22]. Similarly, many phenolic compounds are shown to inhibit secretion and production of proinflammatory cytokines. Although the oregano extracts contain several distinct flavonoids including quercetin, luteolin, and scutellarein glycosides, it is unclear which flavonoid triggers the bioactivity.

Recently, quercetin has been shown to inhibit* in vitro* production of cyclooxygenase (COX) and lipoxygenase (LOX) which are typically induced by inflammation [23]. The anti-inflammatory effect has been supported by* in vivo* experiments as well. Examples of quercetin's inhibitory qualities include the significant blocking of proinflammatory cytokines in cultured fibroblasts [24]. 10 *μ*M quercetin downregulated the production of COX-2, the Nuclear Factor-kappa B (NF-*κ*B), and NO [25]. 10–25 *μ*M quercetin inhibited the level of NO and TNF-*α* [26]. Other properties of 50 and 100 *μ*M quercetin include reducing the secretion of IL-6 and TNF-*α* in LPS-stimulated RAW 264.7 microphages [27]; while at 25 and 50 *μ*M it proved to be the most efficient blocker of TNF-*α* secretion in macrophages. Finally, at low concentrations, quercetin (less than 50 *μ*M) also stimulated anti-inflammatory cytokine IL-10 [28]. Similarly, 25 *μ*M quercetin blocked IL-1*β*, IL-6, IFN-*γ*, and TNF-*α* secretion in human whole blood induced by LPS [29]. Meanwhile, by inhibiting NF-*κ*B activation, quercetin at less than 10 *μ*M inhibited the production of NO, IL-6, monocyte chemoattractant protein-1 (MCP-1), TNF-*α*, iNOS, and COX-2 in RAW 264.7 cells [25]. Quercetin can also inhibit proinflammatory cytokines. A six-week regiment of 150 milligrams of quercetin taken daily by human subjects significantly lowered cytokine TNF-*α* serum concentrations [30]. Quercetin has also been shown to reduce pancreatic histopathological damage and lower the mRNA and protein level of NF-*κ*B, IL-1*β*, IL-6, and TNF-*α* in rats [31].

Despite the extensive evidence for quercetin's anti-inflammatory effect, the mechanism for its success is not well understood. Potential influences could be the inhibition the molecular level of COX-2 and iNOS, NF-*κ*B, AP-1, or mitogen-activated protein kinase (MAPK) [32]. Interrupting these enzymes would have anti-inflammatory impact. NO, a proinflammatory mediator, is synthesised by iNOS due to the reactions by proinflammatory compounds like LPS. Furthermore, other studies have demonstrated that the pretreatment with quercetin inhibits iNOS and NO production induced by LPS and counteracts the oxidative stress created by the unregulated NO production [33]. Meanwhile, NF-*κ*B and AP-1, both inhibited by quercetin, are significant transcriptional features in modulating proinflammatory cytokines [34–36]. In rat aortic endothelial cells, quercetin significantly reduces the production of NF-*κ*B and AP-1 activity [37]. Quercetin has also proven to be an effective pretreatment to combat apoptosis cell death. In addition, quercetin would inhibit phosphorylation of stress-activated protein kinases (JNK/SAPK) and the p38 MAPK, which are responsible for the inhibitory effect of cell growth. The wide array of evidence points to quercetin as potentially a powerful weapon in the fight against the inflammatory disease. It could also potentially prove useful to cells involved in allergic inflammation [38].

## 6. Antiobesity Effect of Quercetin

Research suggested that quercetin downregulated adipogenesis and apoptosis by decreasing the action of adipogenesis-related enzymes; meanwhile, levels of MAPK as well as its substrate acetyl-CoA carboxylase (ACC) were upregulated [39]. Simultaneously apoptosis was induced and a drop in the levels of ERK and JNK phosphorylation was seen. The implication is that quercetin works to block adipogenesis actions through stimulating the MAPK signal pathway. At the same time quercetin induced the apoptosis of mature adipocytes by controlling the important ERK and JNK pathways.

Other authors have shown a role for quercetin in the regulation of hepatic gene expression and lipid metabolism [40]. Studies showed that quercetin prevents high-fat diet (HFD) induced obesity in C57B1/6 mice, perhaps by regulating lipogenesis. Mice fed a quercetin supplement saw a significant lowering of HFD induced obesity compared to those fed the HFD without the supplement. Specifically, the supplement-fed mice experienced a reduction in body weight, liver weight, and amount of total white adipose tissue. Quercetin appears to have modified the gene profiles of genes related to lipid metabolism including Fnta, Pon1, Pparg, A1dh1b1, Apoa4, Abcg5, Gpam, Acaca, Cd36, Fdft1, and Fasn.

## 7. Antidiabetic Effect of Quercetin

The antidiabetic qualities of quercetin involve the stimulation of glucose uptake through an MAPK insulin-dependent mechanism. Stimulation of the mechanism in skeletal muscles has resulted in the translocation of glucose transporter 4 (GLUT4). This role for MAPK is distinct from its role in the liver where it reduces the production of sugar mostly through the downregulation of the key gluconeogenesis enzymes [41].

Studies have been conducted on effects of quercetin in animals with type 2 diabetes [42]. Those that received quercetin showed lower glucose plasma levels relative to the control group and do not experience increase or decrease in insulin measured by the homeostasis model. Animals that received a 0.08% portion of quercetin showed a variety of other improvements including an increase in plasma adiponectin and HDL-cholesterol, decreases in plasma total cholesterol and plasma triacylglycerols, and increases in specific liver enzymes activities important in the detoxification processes. Quercetin has further been shown to play a role in improved renal functioning in diabetic nephropathic rats by blocking the overexpression of connective tissue growth factor (CTGF) and transforming growth factor-*β*1 (TGF-*β*1). End-stage renal disease is closely associated with diabetic nephropathy. Studies show that TGF-*β*1 and CTGF have an essential impact on the DN pathophysiological systems involved. Studies examined the impact of quercetin on TGF-*β*1 and CTGF renal functions in streptozotocin- (STZ-) induced diabetic Sprague-Dawley rats [43]. Results showed that rats treated with quercetin saw a reduction in their weight ratio of kidney and body. The expressions of CTGF and TGF-*β*1 are higher in the renal tissues. For those that received quercetin, the overexpression was reduced.

Finally, quercetin has been shown to produce an effective* in vitro* block against lens aldose reductase and additionally prevents polyol accumulation [44]. For humans, quercetin has shown to help decrease the seriousness of numbness, jolting pain, and irritation for patients with type 2 diabetes neuropathy. It has further been shown that active treatment with quercetin can improve various quality-of-life matrices [45].

## 8. Conclusions

Evidence in various studies seems to connect abdominal obesity, type 2 diabetes, and chronic low-grade inflammation. Researchers have begun to view type 2 diabetes more in terms of inflammation as more confirming evidence has been found. Adipose tissue appears to create changes in cellular composition and the production of proinflammatory cytokines and chemokines. Through an increase of antioxidative activities, NF-*κ*B regulation, the reduction of proinflammatory enzymes activity, and the reduction of cytokine levels, quercetin has shown itself to be a strong anti-inflammation weapon. These positive results have been found in both animal and human studies and support the use of quercetin in fighting inflammatory disease. Nevertheless, continued evaluations are needed to uncover the exact mechanisms through which quercetin functions in order to satisfactorily address safety concerns. It is hoped this study will reignite interest in the anti-inflammatory properties of quercetin and encourage the public to explore vegetarian diets and natural medicines.
